# Simultaneous, Dual Continuous Venovenous Haemodiafiltration as Salvage Therapy for Severe Sodium Valproate Intoxication

**DOI:** 10.1155/2024/2712480

**Published:** 2024-05-06

**Authors:** Aminah Hussan, Ifrah Hasan, Reem El-Hayani, Moustafa Shebl Zahra

**Affiliations:** ^1^King's College London Guy's Campus, Great Maze Pond, London SE1 1UL, UK; ^2^Queen Elizabeth The Queen Mother Hospital, Ramsgate Rd, Margate CT9 4AN, UK

## Abstract

Sodium valproate overdose leads to CNS depression, cerebral oedema, and severe metabolic acidosis in cases of severe toxicity. Extracorporeal removal, specifically through intermittent haemodialysis, is recommended, though not always tolerated by or accessible to haemodynamically unstable patients in intensive care units. We present a case of a male in his mid-twenties presenting following a massive, intentional overdose of 13 g of sodium valproate over 7 hours, with an initial valproate blood concentration of 975 *μ*g/ml (normal 50-100 *μ*g/ml). He was hypoxic and severely acidotic on arrival and was given fluids and L-carnitine according to TOXBASE guidelines. This resulted in only marginal improvement to his acidosis. Once transferred to our intensive care unit, the patient was started on inotropic support followed by continuous venovenous hemofiltration (CVVHDF) at the maximum effluent rate of 60 ml/kg/hr. Due to his persisting metabolic acidosis and worsening hyperlacataemia, dual CVVHDF was started by adding another filter in series after 26 hours, increasing the maximum effluent rate to 96 ml/kg/hr. The patient remained on dual CVVHDF for 31 hours, during which his acidosis and lactate showed considerable improvement, and he was subsequently stepped down to single-filter CVVHDF for a further 20 hours until complete resolution of his acidosis. This case report recognises dual CVVHDF as a viable salvage therapy for severe sodium valproate overdose by facilitating the achievement of a higher effluent flow rate compared to what can be accomplished with single-filter CVVHDF.

## 1. Introduction

Sodium valproate (VPA) is a commonly prescribed antiepileptic drug with a narrow therapeutic range of 50-100 *μ*g/ml [1]. Transgressing this range leads to a reduction in protein binding with a resultant increase in free valproic acid [2], as well as an increased half-life of >30 hours [3]. In patients suffering from a massive overdose, toxicity mostly manifests with central nervous system depression, seen through coma and cerebral oedema [4], and may also result in other life-threatening complications such as metabolic acidosis [5].

VPA-induced toxicity is traditionally managed supportively through airway protection and gastrointestinal decontamination, often supplemented with administration of L-carnitine for hepatotoxicity and hyperammonaemia [6]. In severe overdose, VPA is moderately dialyzable so extracorporeal removal (ECTR) is recommended [7]. Although intermittent haemodialysis (IHD) is the preferred method of elimination [8], it is often not tolerated by, or even accessible to, haemodynamically unstable patients in the intensive care setting. We highlight our management using dual-filter continuous venovenous haemodiafiltration (CVVHDF) to successfully treat a patient with a massive overdose presenting with a profoundly high VPA concentration.

## 2. Case Presentation

A mid-twenties male (60 kg) presented to the emergency department (ED) of our local hospital by ambulance 7 hours after a deliberate overdose of 65 tablets of sodium valproate 200 mg (13 g total). He was found by the ambulance team to be stiff and rigid, with both urinary and faecal incontinence, but no obvious injuries. Notable past medical history includes an ADHD diagnosis and multiple previous suicide attempts.

He presented hypotensive with a BP of 110/80 mmHg, tachycardic with a heart rate of 100/min, and SpO2 averaging 97% on room air. After admission to the resuscitation bay, his initial valproate level was 975 *μ*g/ml. His venous blood gas results showed a metabolic acidosis (pH 7.22) with hyperlactatemia (6.6 mmol/l). He was resuscitated with intravenous fluids and administered treatment doses of L-carnitine and N-acetylcysteine as per the TOXBASE (National Poisons Information Service) guidelines. However, repeat blood gas results after 6 hours showed only marginal improvement in his acidosis (pH 7.29).

In the subsequent hours, his condition worsened, and he was transferred to our ICU. There, he was electively intubated owing to agitation, worsening sensorium, and acidosis. Additionally, he was given inotropic support with a noradrenaline and vasopressin infusion.

**Figure 1:**
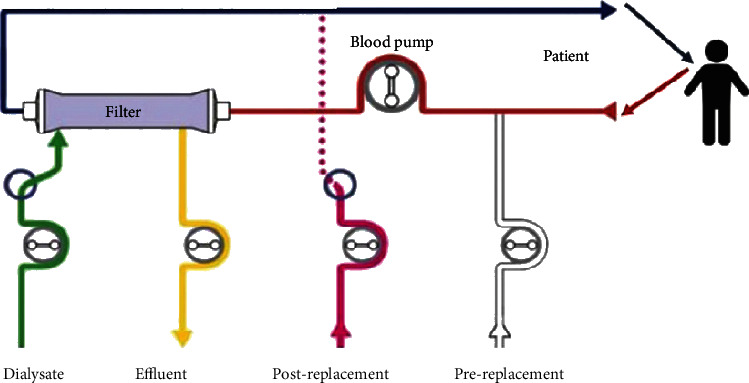
Basic continuous venovenous haemodiafiltration (CVVHDF) configuration taken from East Kent Critical Care Guidelines. CRRT fluid was added to the circuit in three locations: prefilter replacement fluid (added to patients' blood), dialysate (runs on the other side of the filter membrane to the patients' blood), and postfilter replacement fluid (added to patients' blood). The desired total effluent dose was calculated and then divided between dialysate and replacement fluid. Citrate was used for anticoagulation.

On day 1 of ICU, a decision was made to start CVVHDF with one filter at a maximum effluent flow rate of 60 ml/kg/hr (see diagram in Figure 1). He was also treated with L-carnitine as per TOXBASE guidelines. After 24 hours of single-filter CVVHDF, his serum valproate level reached the therapeutic range (shown in Table 1). Yet, his noradrenaline requirements continued to rise, and successive blood gas results revealed a worsening acidosis with increasing lactate levels.

After 26 hours, a second CVVHDF machine was started at an effluent flow rate of 24 ml/kg/hr, which was titrated up to 36 ml/kg/hr achieving a total effluent dose of 96 ml/kg/hr (see Table 2 for full details). The rationale for this was to achieve rapid lactate clearance and subsequently reverse the acidosis so vasoactive drugs could take effect.

**Figure 2:**
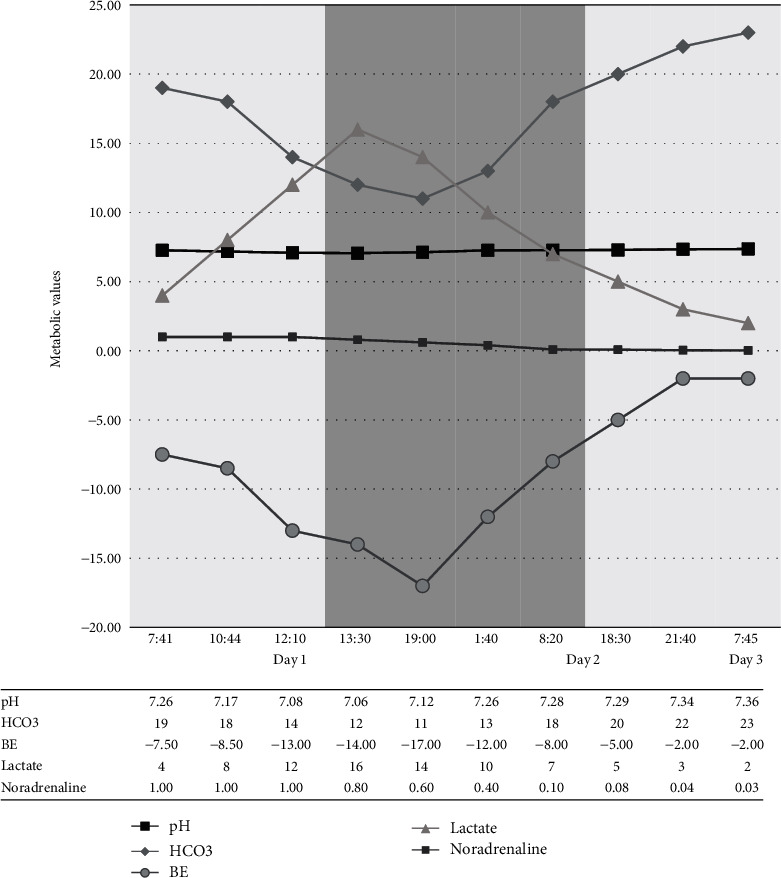
Monitoring biological parameters after initiating continuous venovenous haemodiafiltration (CVVHDF). The graph shows relative dynamic changes to pH, plasma bicarbonate, base excess, lactate, and concentrations of noradrenaline infusion after single-filter CVVHDF is started followed by dual-filter CVVHDF, and finally single-filter CVVHDF.

The patient experienced mild hypotension with the initiation of a second filter causing a slight increase in the noradrenaline requirements. This effect was negligible since the noradrenaline requirements subsequently reduced following the correction of the acidosis. No other complications related to dual-filter CVVHDF were noted. Within 24 hours of dual filtration, successive ABGs showed improved pH and decreased lactate (shown in Figure 2).

Dual CVVHDF was continued for 31 hours, allowing for marked improvement in the patient's acidosis, reduction in lactate levels, and considerable improvement in other biological parameters. A decision was made to discontinue one filter when pH was consistently above 7.2 and a notable reduction in noradrenaline requirements was reached. He remained on single-filter CVVHDF for a further 20 hours until full resolution of his acidosis, ensuring no rebound acidosis occurred before stopping CVVHDF completely. At this time, the patient developed cerebral oedema which was attributed to valproate toxicity. This was successfully managed with hypertonic saline 2.7%.

Despite the resolution of his acidosis, the patient became severely hypoxic and developed acute respiratory distress syndrome (ARDS) on day 4 of his ICU stay.

This was likely related to massive bilateral aspiration pneumonia, with associated bilateral effusions, which had limited improvement despite employing a protective lung strategy.

The patient was eventually transferred for extracorporeal membrane oxygenation (ECMO) at Guy's and St Thomas' Hospital (GSTT) as per protocol. At GSTT, his ECMO management was complicated by a Fournier gangrene which was resolved with subsequent debridement and antibiotic treatment. The patient was repatriated back to our ICU 15 days later and survived with no long-term sequelae. An inpatient psychiatric review was arranged, and the patient was placed under Section 2 of the Mental Health Act (MHA).

## 3. Discussion

To the best of our knowledge, this is the first documented case of severe VPA-induced toxicity successfully managed with dual CVVHDF. Although the EXtracorporeal TReatments in Poisoning (EXTRIP) workgroup recommends IHD over continuous renal replacement therapy (CRRT) for severe VPA overdose, this case report highlights dual CVVHDF as an alternative, potentially superior, management option. This case demonstrates the potential to overcome the limitations of single-filter CVVHDF by employing a second filter in a series.

Given its low molecular weight, (144 Da), low volume distribution, and saturable protein binding nature, VPA is a good candidate for ECTR in severe overdose [9]. There are several reasons that VPA clearance is considered more favourable with IHD compared to CRRT, which includes continuous venovenous haemofiltration (CVVH) and CVVHDF [8]. IHD has a higher clearance rate on average (88 ml/min, reaching up to 140 ml/min), superior clearance of ammonia, and greater capacity to correct acidaemia [7]. This is a high priority in massive VPA overdoses to reverse the acidosis and minimise toxin exposure. CRRT, in comparison, offers improved cardiovascular tolerance and better long-term solute clearances.

There is conflicting evidence on the effectiveness of single-filter CVVHDF to treat VPA toxicity. Whilst several unsuccessful attempts have been reported, a few studies have proven it to be successful in improving metabolic acidosis in haemodynamically unstable patients with severe VPA overdose [10, 11].

Ge et al. documented a case of survival from VPA intoxication following treatment with fractionated plasma separation and adsorption mode integrated with continuous venovenous haemofiltration (FPSA-CVVH), typically used for liver support therapy. They found that FPSA-CVVH provided an option for patients unable to endure IHD due to their haemodynamic status, plus offered the additional benefit of effectively removing protein-bound VPA under the therapeutic dose [12].

Moreover, CVVHDF following in-series haemodialysis and haemofiltration has been shown to prevent postdialytic rebound whilst first optimising drug clearance in a case of severe VPA toxicity [13].

Previously documented cases of dual continuous haemofiltration have shown positive outcomes for overdose-induced lactic acidosis (see Supplemental Table S1). In their management of metformin-induced lactic acidosis, Seube et al. used two simultaneous CVVH machines to yield a total haemofiltration dose of 98 ml/kg/hr. Plasma metformin was normalised after 32 hours of dual CVVH. Similar to us, this case report mentioned that IHD was unavailable in their ICU and likely not tolerated by their patient [14].

Reynolds et al. reported a series of instances involving severe metformin overdoses, which were effectively treated using a dual CVVHDF approach, resulting in a total haemofiltration dosage ranging from 57 to 165 ml/kg/hr [15]. Their overall finding was that dual CRRT effectively doubles the blood volume and extracorporeal circuit flow rate. However, they acknowledged that this might partially diminish the advantage of single CRRT in terms of haemodynamic stability compared to IHD. Nonetheless, we demonstrated that this effect can be effectively managed by initiating the dual filters sequentially.

To conclude, this case underscores dual CVVHDF as a rescue therapy for severe VPA overdose in haemodynamically unstable patients admitted to ICU. The ICU staff's familiarity with this technique offers a further advantage to this. This technique allows the total haemofiltration dose to be increased which primarily helps treat the metabolic acidosis and hold these patients for longer, and secondly, effectively clears protein-bound VPA.

## Figures and Tables

**Table 1:** Serum VPA level on admission then after clearance with continuous venovenous haemodiafiltration (CVVHDF). The initial 24 hours of treatment was with single-filter CVVHDF. At 48 hours, there had been 22 hours of dual-filter CVVHDF following an initial 26 hours of single-filter CVVHDF. Finally, dual filtration was stepped down to single filtration after 31 hours. Single-filter CVVHDF was continued for a further 20 hours.

	Serum VPA level (*μ*g/ml)
On admission	975
After CVVHDF treatment	
24 hrs	82
48 hrs	23
77 hrs	<13

**Table 2:** Settings for each continuous venovenous haemodiafiltration (CVVHDF) machine.

Dialysis parameters	CVVHDF 1	CVVHDF 2
Weight (kg)	60	—
BFR (ml/min)	150	100
PBP Cit (mmol/l)	3.0	3.0
PBP (ml/hr)	1500	100
Dia (ml/hr)	1500	900
Rep (ml/hr)	600	300
Ci load	14.7	10.2
Total effluent (ml/kg/hr)	60	36

BFR: blood flow rate (in ml/min); PBP Cit: concentration of citrate within the blood (mmol/l of blood); PBP: preblood pump (flow rate of citrate in ml/hr—calculated by machine when PBP Cit specified); Dia: dialysate flow rate (in ml/hr); Rep: postfilter replacement fluid (haemofiltration/convective part of RRT in ml/hr); Ci load: calculated citrate load (in mmol/hr) based on haematrocrit 30% (should be <30 mmol/hr).

## Data Availability

There is no data availability to be reached. All the information is supplied in the manuscript.

## Supplementary Materials

Supplementary Materials Table S1: Previously documented cases of dual continuous renal replacement therapy (CRRT) to treat overdose-induced lactic acidosis.
