# Supplementary material for: Simultaneous, Dual Continuous Venovenous Haemodiafiltration as Salvage Therapy for Severe Sodium Valproate Intoxication
Source: Case Rep Crit Care. 2024 May 6;2024:2712480. doi: 10.1155/2024/2712480 (PMC11090666; doi:10.1155/2024/2712480)
Supplement: Supplementary Materials — Table S1: Previously documented cases of dual continuous renal replacement therapy (CRRT) to treat overdose-induced lactic acidosis. [file 2712480.f1.docx]

**Table S1** – Previously documented cases of dual continuous renal replacement therapy (CRRT) to treat overdose-induced lactic acidosis

| **Author, year of publication** | **Reynolds et al. (2022)** | | | | **Seube et al. (2023)** |
| --- | --- | --- | --- | --- | --- |
| **Case** | **1** | **2** | **3** | **4** |  |
| **Weight (kg)** | 61 | 110 | 72 | 118 | 102 |
| **Primary drug intoxication** | Metformin | Metformin | Metformin | Metformin | Metformin |
| **Initial serum lactate (mmol/L)** | 4.3 | 2.3 | 18.2 | 11.3 | 27.4 |
| **Peak serum lactate (mmol/L)** | 29.2 | 22.2 | 28.9 | 18 | 27.4 |
| **Mode** | CVVHDF-CVVHDF | CVVHDF-CVVHDF | CVVHDF-CVVHDF | CVVHDF-CVVHDF | CVVH-CVVH |
| **Total time of single CRRT prior to starting dual CRRT (hr)** | 4 | 12 | 3 | 9 | 11 |
| **Reason for starting dual CRRT** | Poor clearance of lactate | Poor clearance of lactate | Haemodynamic instability associated with increase in noradrenaline requirement | Rising lactate concentrations | Poor clearance of lactate in addition to haemodynamic instability associated with increase in noradrenaline requirement |
| **Total time for dual CRRT (hr)** | 33 | 10 | 8 | 10 | 32 |
| **Total haemofiltration dose with dual CRRT (ml/kg/hr)** | 165 | 76 | 156 | 57 | 98 |
| **Complications associated with CRRT** | None reported | Persistent hypotension | First filter clotted but there was no longer need for dual filter since pH and lactate and normalised; then second filter clotted but there was no longer need for ongoing dialysis | None reported | None reported |
| **Patient survival** | Yes | No (death from multi-organ failure; delay in starting second filter CVVHDF meant normalisation of pH was not achieved) | Yes | Yes | Yes |
